# Increased c-MYC Expression Associated with Active IGH Locus Rearrangement: An Emerging Role for c-MYC in Chronic Lymphocytic Leukemia

**DOI:** 10.3390/cancers16223749

**Published:** 2024-11-06

**Authors:** Kenza Guiyedi, Milène Parquet, Said Aoufouchi, Jasmine Chauzeix, David Rizzo, Israa Al Jamal, Jean Feuillard, Nathalie Gachard, Sophie Peron

**Affiliations:** 1 Centre National de la Recherche Scientifique (CNRS), Unité Mixte de Recherche (UMR) 7276/INSERM U1262, Université de Limoges, 87000 Limoges, France; 2 Gustave Roussy, B-Cell and Genome Plasticity Team, CNRS UMR9019, Villejuif, France and Université Paris-Saclay, 91400 Orsay, France; 3 Laboratoire d’Hématologie Biologique, Centre Hospitalier Universitaire de Limoges, 87000 Limoges, France; 4 Faculty of Sciences, GSBT Genomic Surveillance and Biotherapy Team, Mont Michel Campus, Lebanese University, Tripoli 1300, Lebanon

**Keywords:** MYC, CLL, recombination, DNA repair, B cell

## Abstract

This review examines the pivotal role of c-MYC in Chronic Lymphocytic Leukemia (CLL), focusing on how its overexpression leads to increased genetic instability, thereby accelerating disease progression. *MYC*, a major oncogene, encodes a transcription factor that regulates essential cellular processes, including cell cycle control, proliferation, and apoptosis. In CLL cases enriched with unmutated immunoglobulin heavy chain variable (IGHV) genes, *MYC* is significantly overexpressed and associated with active rearrangements in the *IGH* immunoglobulin heavy chain locus. This overexpression results in substantial DNA damage, including double-strand breaks, chromosomal translocations, and an increase in abnormal repair events. Consequently, c-MYC plays a dual role in CLL: it promotes aggressive cell proliferation while concurrently driving genomic instability through its involvement in genetic recombination. This dynamic contributes not only to CLL progression but also to the overall aggressiveness of the disease. Additionally, the review suggests that c-MYC’s influence on genetic rearrangements makes it an attractive target for therapeutic strategies aimed at mitigating CLL malignancy. These findings underscore c-MYC’s critical importance in advancing CLL progression, highlighting the need for further research to explore its potential as a target in future treatment approaches.

## Simple Summary

This review explores the role of c-MYC in Chronic Lymphocytic Leukemia (CLL) and its impact on genetic instability and disease progression. As a key oncogene, c-MYC encodes a transcription factor involved in regulating cell cycle, growth, and apoptosis. We previously described CLL cases enriched with unmutated IGHV genes, *MYC* overexpression and with active rearrangement of the *IGH* immunoglobulin heavy chain (IGH) locus. The *MYC* overexpression seems to promote increased DNA damage, including double-strand breaks (DSBs), chromosomal translocations linked to repair errors during DNA repair. Highlighting c-MYC’s dual role, this review try to show how *MYC* overexpression it not only driving cell proliferation but also contributes to genomic instability.

## 1. Introduction

Chronic Lymphocytic Leukemia (CLL) is a highly frequent B-cell cancer, mostly affecting the elderly. With a very variable clinical presentation, the overall survival of CLL patients ranges from years to decades and remains incurable. CLL is an indolent lymphoma characterized by the accumulation of small monomorphic round CD19+ CD23+ CD5+ B lymphocytes (B-cells). CLL diagnosis is based on the detection of monoclonal CLL cells in the blood with lymphocytosis exceeding 5 g/L and infiltration of lymphoid organs. There is a preneoplastic stage termed monoclonal B-cell lymphocytosis (MBL) characterized by a blood clonal B-cell count <5 g/L without tumor infiltration of tissues or any feature diagnostic of another B-cell lymphoma or disorder. When CLL cells infiltrate secondary lymphoid organs, leading to lymphadenopathy or splenomegaly and lymphocytosis is <5 g/L in the blood, the diagnosis leads to small lymphocytic lymphoma (SLL) [1]. After CLL diagnosis, patient care and clinical management are decided based on Rai or Binet staging classifications [2,3]. Both classifications, easy to use for physicians, reliably predict disease progression. Moreover, karyotype and genetic heterogeneity influence overall survival and therapeutic response. The CLL International Prognostic Index (CLL-IPI) classifies patients by combining Rai and Binet scores with genetic parameters and provides treatment recommendations [4]. Several chromosomal abnormalities are recurrent in CLL and are predictive of CLL progression and/or resistance to therapy. Among these recurrent abnormalities, the most frequent and/or important in the decision tree for treatment include deletion of the long arm of chromosome 13 (del (13q)), trisomy 12, and other markers of poor prognosis, which are deletion of the long arm of chromosome 11 (del (11q)) and deletion of the short arm of chromosome 17 (del (17p)). Other poor prognosis genetic abnormalities involve the Notch pathway (*NOTCH1* mutations), NF-kappa B activation (*BIRC3* or *MYD88* mutations), splicing (*SF3B1* mutations), the DNA lesion sensor *ATM* or the *TP53* anti-oncogene (mutations and/or deletion) [4]. In addition, since the publication of papers by Hamblin and Damned [5,6], it is globally accepted that the mutational status of genes coding for the variable (*IGHV*) part of the immunoglobulin (Ig) heavy-chain (IGH) locus is an indicator of CLL prognosis. CLL patients with an unmutated variable region (umCLLs) have a poor prognosis, while patients whose IGHV gene has been targeted by mutations (mCLL) have a higher survival rate. Recently, we described CLLs with poor prognoses characterized by high c-MYC expression associated with active *IGH* locus recombination [7]. An increase in *IGH* recombination induced by MYC overexpression was observed in the CH12F3 B lymphocyte cell line, confirming that c-MYC potentiates the rearrangement of the IGH locus. Nevertheless, the precise mechanism remains questionable, and in this review, we recapitulate data from the literature concerning c-MYC to discuss the potential modus operandi and impact of c-MYC-induced *IGH* recombination on tumoral CLL B-cells.

## 2. c-MYC, a Master Orchestrator of B Cell Fate

**Figure 1:**
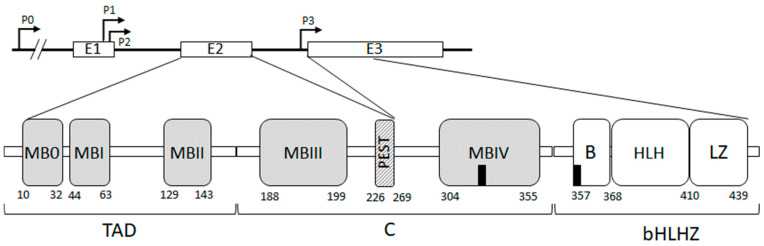
*MYC* gene and protein: the gene contains 4 promoters (P) and 3 exons (upper diagram). The protein contains 3 domains (lower diagram): the N-terminal transactivation domain (TAD) includes 3 conserved MYC boxes: MB0, MBI, and MBII. The Central domain (C) includes an acidic PEST domain, a MYC box IV, and a nuclear Localization site, NLS (black square). The C-terminal b-HLHZip domain includes a basic region (B), which contains a second NLS, a helix-loop-helix (HLH), and a leucine zipper (LZ). Not drawn to scale (from [15,16,17,18]).

The human MYC proto-oncogenes encode a family of three transcription factors: c-MYC, N-MYC, and L-MYC, encoded by genes located on chromosomes 8, 2, and 1, respectively. These MYC paralogs have similar structural architectures and functions but differential patterns of expression with distinct times and locations of expression during cell differentiation. The *MYC* oncogene was discovered because of its homology with the v-MYC oncogene in avian acute leukemia virus (MC29) [8]. More precisely, the c-MYC oncogene is located at 8q24 [9] and is composed of three exons (Figure 1): the non-coding exon 1 and exons 2 and 3 encoding the c-MYC transcription factor. In humans, four *MYC* promoters have been identified: P0 (human-specific and representing 5% of transcripts) [10], P1 (10 to 25% of transcripts), P2 (75 to 90% of transcripts) [11], and P3 (5% of transcripts) [12].

c-MYC protein is composed of 439 amino acids and is subdivided into three domains (Figure 1). The N-terminal transactivation domain (TAD) (residue 1–143), necessary for the biological activity of c-MYC and c-MYC mediated transcriptional activation, contains three short conserved segments, termed MYC boxes (MB0, MBI, and MBII). MBI, by recruiting cyclin CDK complexes, promotes transcriptional elongation by RNA polymerase II (RNAPII) phosphorylation [13]. MBII is required for c-MYC targeting of transcriptional activation and repression and controls c-MYC turnover. The central domain includes an acidic PEST domain (residues 226–229) adjacent to MBIII and MBIV conserved boxes. MBIII can recruit the histone deacetylase 3 (HDAC3) and is engaged in transcriptional repression. MBIV contains the first nuclear localization site (NLS1, aa 320–328) and is required for c-MYC pro-apoptotic function. The C-terminal domain consists of a basic region that contains the second nuclear localization site (NLS2, aa 364–374), a helix-loop-helix (HLH) domain, and a leucine zipper. The bHLH-Zip domain forms stable heterodimer c-MYC/Max that directly binds DNA sequences at the canonical Enhancer box (E_box, 5′CACGTG-3′) to stimulate transcription [14].

The oncoprotein c-MYC is a ubiquitous transcription factor that regulates 10–15% of all human genes [19]. The ability of this protein to bind the consensus sequence (CACGTG) of gene E-boxes [20] results in a broad field of action and a key role in cell homeostasis, participating in the regulation of cell cycle, cell growth, survival, cellular metabolism, etc. [21,22], and also suggests a strict transcriptional regulation [23]. As MYC factors exert fundamental roles in cell functions in all tissues, they play a central role in B-cells, starting at B lymphoid lineage establishment to terminal differentiation into memory B-cells and plasma cells.

During early B-cell development, c-MYC regulates B-cell proliferation and survival depending on the developmental stage (Table 1). The crucial role of c-MYC in B-cell progenitors has been demonstrated using a mouse model of c-MYC conditional inactivation in B-cells, which showed a significant increase in apoptosis at all stages of early B development as well as the requirement for c-MYC in cell proliferation with a specificity depending on each developmental stage [24,25]. The expansion of pro-B cells has been shown to depend on the opposing effects of EBF1 and PAX5 on *MYC* gene regulation [26]. Presumably, c-MYC regulation relies on the balance between these two transcription factors, switching from negative to positive regulation in the same network, and also depends on the response to cytokines, including IL-7 [24]. In pre-B cells, c-MYC inactivation arrests cell development and blocks differentiation in immature B-cells. Specifically, during the maturation and expansion of large pre-B cells into small pre-B cells, c-MYC and *N-MYC* are expressed. In immature B cells, only c-MYC is expressed and then, in mature B cells, c-MYC is expressed only in mature B cells after their activation [27].

Following bone marrow exit, naïve mature B lymphocytes express a complete BCR and enter the peripheral immune system. In primary B cells, c-*MYC* expression induces an increase in intracellular calcium, which is itself necessary for stimulation of B cell proliferation and differentiation by c-MYC, as shown by Habib et al., 2007. c-MYC reduces PMCA4, which promotes proliferation, differentiation, and immunogenic response of B cells dependent on NFAT [27]. Furthermore, constitutive c-MYC signaling alone or supported by calcium/NFAT signals promotes B cell activation/differentiation or tumorigenesis. Finally, these authors also reported that *MYC* expression inducing high calcium levels increased NFAT translocation in the absence of upstream signaling from pre-BCR/BCR and increased the short isoform level of NFAT (NFATc1A), which does not promote apoptosis in lymphocytes compared to other NFATc1 isoforms of. Therefore c-MYC inducing high levels of calcium in B cells influences transcriptional and biological responses in a major fashion. In peripheral B cells, Habib and al. report that deletion of c-MYC and N-MYC together decreases peritoneal B cells, which express CD5^low^, IgM^high^ (B1 B cells), and conventional B cells (CD5^neg^, IgM^high^, B2 B cells) suggesting that c-MYC is essential for the development of both populations. Antigen (Ag) binding by the BCR provides signals to B-cells to relocalize in the secondary lymphoid organs. Depending on the Ag nature, B-cells undergo T-cell independent or dependent activation. Follicular B-cells confronted with co-activation by CD4+ T lymphocytes and antigen-presenting cells will be activated. This process triggers class switch recombination (CSR) and germinal center (GC) formation, allowing the generation and selection of B-cells able to produce high-affinity antibodies. GC is a dynamic structure defined by dark (DZ) and light (LZ) zones, which are both characterized by differential marker expression at the surface of B-cells and stromal cells and different cellular compositions of the microenvironment. In the GC, B cells proliferate strongly: at this stage, B-cells are called centroblasts and undergo somatic hypermutation (SHM) of their immunoglobulin genes in the DZ. Conversely, in the LZ, B-cells, the centrocytes, reduce their proliferation and undergo cell selection dependent on Ag-BCR affinity. Selected cells generate plasma cells after terminal differentiation or memory B cells. B-cells with low BCR affinity for Ag enter either apoptosis or a state of energy. These cells can also be redirected to the DZ for a new cycle of selection. In order to control and regulate the GC reaction, different molecular pathways are involved. c-MYC represses the cell cycle inhibitor p-27 and, to a lesser extent, p-21 and is essential for the initiation and maintenance of the GC process [28]. In the DZ, where B-cell proliferation occurs at a high rate, BCL6 is expressed in order to protect the cell from apoptosis and to induce proliferation. c-MYC determines the number of cell divisions that occur in the DZ and is essential in GC initiation, maintenance, and positive selection [29,30,31,32,33,34,35]. In the LZ, CD40 and BCR activation signals induce increased PI3K/AKT pathway signaling, which in turn induces *MYC* expression [29,36]. This is also supported by the NF-κB and mTORC1 pathways. It has been shown that *MYC* expression is quantitatively proportional to the strength of T cell help in the LZ [30]. Cells that have actively received help from T cells promote the development of plasma cells and limit differentiation into memory cells [32,37,38]. The transcriptional program switch from B-cells to plasma cells is governed by BLIMP1, which suppresses *MYC* expression [28,31]. During the transit between the LZ and DZ, *MYC* and *BCL6* are both expressed and will be regulated depending on other zone-specific regulatory factors.

As previously mentioned, c-MYC is an important player in B lymphocyte development. Its expression is induced in waves depending on the stage, and its activity has been particularly implicated in the proliferation and apoptosis of B cells, modulated by other factors regulating these mechanisms.

## 3. c-MYC Implication in B Cell Cancers

Cancer Genome Atlas reports that c-MYC and its paralogs are involved in 28% of cancers [39]. c-MYC deregulation is more prevalent than N-MYC and L-MYC in human cancer types for both hematological and solid tumors [40]. Different mechanisms drive abnormal *MYC* activation in human cancers. This can be achieved through genetic alteration, gene expression deregulation, or protein modification (Table 2). The first implication of c-MYC in human cancer arose with the identification of the *MYC* gene translocation to the *IGH* locus in human Burkitt lymphoma (BL) [11]. c-MYC plays a role in several types of B-cell acute leukemia: B lymphoblastic leukemia (B-ALL) with t(9; 22) and *BCR::ABL1* rearrangement, B-ALL with t(v; 11) *KMT2A* rearrangement, B-ALL with the t(12; 21) and *ETV6::RUNX1* rearrangement and B-ALL with other chromosomal rearrangements as *TCF3*::*PBX1* in leukemia derived from pre-B lymphocytes [24]. In the last WHO revision of the classification of tumors of hematopoietic and lymphoid tissues, there is a subtype with *MYC* rearrangement distinct from other B-ALL types and Burkitt lymphoma (ref OMS 2022). c-MYC is also involved in many germinal center-derived lymphomas and the histological transformation of indolent mature B-cell malignancies (follicular lymphoma, CLL, MALT, DLBCL, BL, and PBL) [24,31].

In precursor B cells, BCL2 cooperates with c-MYC to promote their proliferation, but this cooperation can allow these cells to become tumorigenic, as shown by Strasser et al., 1990 when using double transgenic Eµ-Bcl2/myc mice, which developed more tumors compared to Eµ-myc only mice [41]. Bissonnette et al., 1992 and Fanidi et al. 1992 also demonstrated the inhibition of apoptosis mediated by MYC of BCL2 in some cells but not the proliferative function of c-myc [42,43]. Other proteins cooperate with c-MYC in tumorigenesis such as RAS, MYB, PIM1, RAF1 [44].

Its role in B lymphomagenesis begins in hematopoietic malignancies, where genomic abnormalities involving the *MYC* gene are almost always found. In Burkitt lymphoma, *MYC* rearrangement with one of the *IG* gene loci is a genetic hallmark of this lymphoma. In 80% of cases, *MYC* translocation will be with the *IGH* locus in 14q32 or with *IGK* or *IGL* genes at 2p12 or 22q11 in 10% of cases each [24,45]. In DLBCL, the c-MYC protein is detected in approximately 40% of patients at diagnosis without gene alterations and its rearrangement is found in approximately 10% of DLBCL cases [24,31,33,45]. As for Burkitt’s lymphoma, Ig genes are the most frequent *MYC* partners, but they can also translocate in 35–50% with *BCL6*, *BCL2*, *PAX5,* or even *IKZK1* [24]. In follicular lymphoma, up to 75% of histological transformations to a high-grade lymphoma (t-FL) show *MYC* mutations/translocations/amplification or gains associated with *MYC* overexpression and other alterations. In PBL, recurrent somatic mutations in the gene encoding *BLIMP1* (*PRDM1*) occur in 50% of cases, and in 80% of diagnoses, c-MYC and BLIMP1 proteins are co-expressed. As in BL and DLBCL, *MYC* rearrangements are also characteristic of an aggressive subset in PBL. Along with *MYC* gains, they are found in ~50% of cases as partners and in ~85% of cases, the *IG* genes with t(8; 14) *MYC*::*IGH* as common fusion product. c-MYC pathway deregulation is also one of the major events in MALT transformation to DLBCL in 40–80% of cases. R.L. Brinster’s group was the first to highlight *MYC* implication in tumoral transformation using a mouse model expressing *MYC* under the dependence of Eµ or Ig enhancers [46]. This work showed that abnormal *MYC* expression induces B-cell lymphomagenesis resulting in aggressive B-cell lymphomas. Other transgenic or knock-in mouse models, which expressed the *MYC* oncogene under Eµ/3′RR transcriptional control provided a better understanding of c-MYC-dependent B-cell malignant transformation mechanisms [47].

Benhamou et al. (2018) described an axis composed of c-MYC, a miRNA cluster (miR17-92), and the signaling molecule PTEN, which is critical for balancing PI3K activity in its control of B cell-fate decisions for positive and negative selection of immature B cells [48]. In their study, the authors pointed out that CD19 is a major B Cell Receptor (BCR)-independent regulator of c-MYC levels in B cell neoplasms by activating the PI3K/AKT/GSK3 axis to promote B cell transformation and lymphoma progression [49,50]. The same team also demonstrated the role of the c-MYC/miR17-92/PTEN axis in Recombination Activating Genes (RAG) expression in early B cell development. Indeed, they used different genetically modified mouse models and showed that altered function of the c-MYC/miR17-92/PTEN axis alters the PI3K/AKT/FOXO1 pathway, leading to deregulation of RAG expression and blockage of pre-BCR assembly [48].

**Table 2:** Molecular mechanisms associated with abnormal MYC activation in human cancers.

Type of Abnormalities	Observed Defects	References
Genetic aberrations	Insertional mutagenesis Chromosomal translocations	(Dudley et al. 2002) [51] (Erikson et al. 1983) (Meyer et Penn 2008) [52,53]
Gene expression	Gene amplification Deregulation of pathways that influence *MYC* oncogene transcription	(Collins et Groudine 1982) [54] (Alitalo et al. 1983) [55] (Nau et al. 1985) [56] (Mariani-Costantini et al. 1988) [57] (Münzel et al. 1991) [58] (Augenlicht et al. 1997) (Kalkat et al. 2017) [59,60] (He et al. 1998) (Yagi et al. 2002) [61,62]
Protein modifications	Post-translational modifications of MYC proteins	(Allen-Petersen et Sears 2019) [63] (Sears et al. 2000) [64]

## 4. c-MYC in Chronic Lymphocytic Leukemia

In CLL, *MYC* expression is detectable in proliferating pseudo-follicle CLL cells (>25% of c-MYC-positive cells). Moreover, deregulation of the c-MYC pathway and its gain in activity are frequently observed (>70% of cases). Simultaneous aberrations in *MYC* and *TP53* are very common in Richter’s transformation of CLL. In most cases, the *MYC* gene remains intact [24,31].

Causes of *MYC* over-activation, in addition to its genetic change, are diverse: gain of the miR-17-92 cluster regulating *MYC*, mutations/deletions of *MYC* regulator *MGA*, mutations in *MYC*-transcriptional activator *NOTCH1*, T-cell interactions via CD40L or interactions with stromal or nurse-like cells producing BAFF and activating NF-κB and/or BCR signaling, mutation in BCR signaling related genes (*CD79* or *CARD11*) driving the expression of *MYC* in general. An interesting mechanism of BCR activation leading to *MYC* induction has also been proposed in CLL. IgM signaling promotes increased translation of *MYC* RNA and thus participates in a feedback loop that enhances c-MYC activity by eIF4. In some cases, the Ig surface of CLL cells is completely glycosylated in a constant region and contains mannosylated sites in the variable region. These glycosylated Ig interact with lectins, and dissimilar to the normal surface Ig reaction with antigen, binding to lectins does not cause surface Ig endocytosis but leads to persistent signaling and strong ascending regulation of *MYC*.

Overall, BCR signaling in *MYC*-driven malignant B cells is an advantage, but BCR-mediated signaling can be considered an important but replaceable means of ensuring c-MYC activity. In addition, higher levels of c-MYC are able to affect BCR signaling, including its binding to the promoter regions of CD79a and SYK and their overexpression in precancerous B lymphocytes in *Eμ-myc* mice. High c-MYC levels can also downregulate inhibitors of BCR signaling by regulating microRNAs or even lead to its deregulation by inducing the *miR-17-92* cluster (*miR-19*, *miR-18α*) [65,66,67,68]. In this case, genomic regions containing these miRNAs are frequently upregulated/amplified in Richter’s transformation of CLL and clinically aggressive CLL. Moreover, in *t*-FL, when c-MYC binds to the region upstream of *miR-150*, its level is reduced. *Mir-150* regulates transcription factor FOXP1, and it has been shown that lower *miR-150* levels and higher FOXP1 levels are associated with shorter survival in CLL [31].

Deregulation of BCR signaling is not the only impact of c-MYC-mediated microRNA regulation. Many hallmarks of cancer are impacted such as proliferation, cell survival, angiogenesis, vasculogenesis, metabolism and apoptosis by repression of *miR-15a/16-1*, *miR-28*, *miR-29*, and *miR-34α*, upregulated of *miR-17-92*, *miR-106a/b*, *miR-20a*, *miR-93*, *miR-17*, and *miR-23a/b* or cooperation of c-MYC with *miR-548* and *miR-155* for example. It should be noted that *miR-34α* is also known to be the first link between DNA damage response and BCR signaling by limiting the propensity of the BCR pathway via repression of FOXP1 during the DNA damage response, thus ensuring interruption of this pro-proliferative signal. In addition, in B-cell malignancies, in vivo, induction of *miR-34α* and arresting BCR signaling by using Fludarabine and doxorubicin did not work in malignant cells with impaired p53 pathways. Therefore, the repression of both *miR-150* and *miR-34α* by *MYC*-activating aberrations in combination with *TP53* deletion/mutation, which also represses *miR-34α* and which are both very common in Richter’s transformation of CLL, could lead to prominent BCR signaling upregulation. The down-modulation of both *miR-150* and *miR-34a* has been reported as a constant phenomenon.

## 5. MYC and Treatment for CLL

Up to 2020, immunochemotherapy was the backbone of first-line CLL treatment [69]. Based on fludarabine eligibility, the reference treatment was fludarabine, cyclophosphamide, and rituximab combinaison (FCR) with 6 or 4 monthly cycles depending on age. In FCR-ineligible patients, immunotherapy remained the gold standard using the combinaison of bendamustine and rituximab or chlorambucil and obinutuzumab. The presence of a significant TP53 alteration contraindicates immunochemotherapy, making search tests mandatory before any treatment. When TP53 is altered, continuous ibrutinib treatment is the gold standard. Current guidelines recommend targeted therapies as first-line treatment, mainly BTK inhibitors and venetoclax alone, in combination or combined with obinutuzumab. FCR is no longer proposed as a first-line treatment.

MYC exhibits prognostic significance in chronic lymphocytic leukemia (CLL) concerning two types of 8q24 abnormalities: translocations involving MYC and genomic gains. MYC translocations are infrequent, while gains of one or more copies of the MYC gene occur in less than 1% and 5–6% of CLL cases, respectively. The poor prognosis associated with these alterations correlates with an aggressive clinical course and reduced overall survival (OS) [70]. Additionally, MYC gain may be associated with del (17p), which further exacerbates the prognosis [71]. However, alterations in MYC do not yield specific treatment recommendations, and their impact on therapeutic outcomes remains inadequately characterized. The potential role of MYC activation in mediating drug resistance, particularly to BTK inhibitors, is currently unknown.

Recently, MYC has been identified as a potential therapeutic target in CLL. Targeting the PHB-eIF4F complex inhibits MYC translation, resulting in reduced proliferation and survival of CLL cells. These results suggest that disruption of the PHB-eIF4F interaction could represent a promising therapeutic strategy for CLL patients by down-regulating MYC activity and modifying the malignant phenotype [72]. Furthermore, MYC orchestrates metabolic reprogramming in CLL cells, and a study by Simon-Molas et al. [73] shows that it is possible to target MYC-mediated metabolic alterations to improve therapeutic outcomes in CLL.

## 6. c-MYC Contribution to DNA Damage and Recombination

Under normal conditions in B lymphocytes, waves of *MYC* expression are coordinated with specific development stages, and when *MYC* is expressed, its activity is principally interpreted regarding its role in regulating proliferation and apoptosis. In addition, in CLL and Richter’s syndrome, the contribution of c-MYC to tumorigenesis is related to its role in cell proliferation. In our previous work [7], we observed an association between increased *MYC* expression and an active recombination process at the constant part of the IGH locus in CLL cases. This observation reinforces the concept that c-MYC promotes DNA rearrangement, which can occur through several mechanisms.

First, increased c-MYC-induced proliferation can be the source of non-programmed double-strand breaks (DSBs), leading to genetic rearrangement if misrepaired. Indeed, DNA DSBs incessantly arise in the cell genome. These non-programmed DSBs are mainly produced through the conversion of single-strand breaks (SSB). The latter are produced during replication fork collapse or stalling because of topological stress [74,75,76,77,78,79]. In certain circumstances, replication and transcription proceed on the same genes and collide (TRI for transcription–replication interaction) [80,81,82], resulting in DNA DSBs [83]. Other replication obstacles, such as non-B DNA structures (R loops, G quadruplexes, hairpins, cruciform) or chromatin alteration, can also lead to DNA lesions and breakage. Transcriptional stress has also been described as source a of DSBs. Altogether, with increased cell replication and transcription, it is highly conceivable that increased c-MYC activity can potentialize DNA recombination.

Furthermore, c-MYC may be involved in mechanisms that are not restricted to its transcription factor function-inducing players implicated in cell proliferation, as c-MYC can be involved directly in processes leading to genetic rearrangements. Littler et al. examined the ability of c-MYC to modulate mitosis [84]. The authors developed the Tet-on FC-*MYC* cell model with a tetracycline-sensitive *MYC* transgene and mutated endogenous *MYC* alleles. Thus, they were able to study the role of c-MYC in mitotic chromosome segregation by modulating c-MYC expression either weakly (so-called MYC-Low system) or strongly (so-called MYC-High system). What was observed in MYC-High cells was a change in mitotic timing, as well as a morphological change in the metaphase mitotic spindle. In MYC-High cells, the rupture of the nuclear envelope in metaphase was accelerated, while the transition from metaphase to anaphase was delayed (increased metaphase in MYC-High cells). In addition, automated analysis of high-throughput images revealed a change in the proportions of the mitotic spindle during metaphase in terms of length and width in MYC-High cells (increased width and reduced length). Yet, as Levine and Holland’s review of work identifying the sources of mitotic errors in human tumors and their effect on cell fitness and transformation reminds us, mitosis is a delicate event that must be executed with great fidelity to ensure genomic stability [85]. Data in the literature also argue that c-MYC potentializes DNA breaks, which promote DNA recombination. For example, Vafa et al. reported that overexpression of c-MYC in vitro in resting human fibroblasts led to an increase in reactive oxygen species (ROS) correlated with DNA DSB accumulation, which was not associated with cell entry into apoptosis [86]. The same study showed that cells could enter the cycle despite the presence of DNA damage by overriding P53-mediated cell cycle arrest, leading to the proliferation of damaged cells. On the other hand, Ray et al. [87] demonstrated that c-MYC could also induce widespread DNA breaks both in vitro (in normal human foreskin fibroblasts) and in vivo (in murine lymphocytes) without ROS intervention. Furthermore, Karlsson et al. showed that, in addition to increasing DSB generation in vitro (in normal human fibroblasts inducibly overexpressing c-MYC or NHF-MYCER), c-MYC overexpression led to a global defect in the repair of these lesions by affecting the homologous recombination (HR), non-homologous end joining (NHEJ) and single-strand annealing (SSA) pathways; but not the nucleotide excision repair (NER) pathway [88]. These results were obtained by testing the efficiency of DSB repair in a hamster cell line designed to contain a GFP-coupled recombination substrate and inducible for *MYC* overexpression (DRAA8-MYCER). These same studies also highlighted the role of c-MYC in inducing genetic rearrangements. Indeed, karyotypic analysis of 100 NHF-MYCER metaphases showed that *MYC* overexpression induces chromatid breaks, deletions, and translocations. Finally, Chiang et al. pointed out that Nijmegen syndrome, a rare genetic disorder of chromosomal instability associated with cancer predisposition, radiosensitivity, and chromosomal instability, involves the NBS1 (p95 or nibrin) gene, part of the MRE11/RAD50/NBS1 complex which is an essential component of the NHEJ repair system [89]. In vitro, in lymphoblastoid cell lines overexpressing c-MYC either constitutively (CBMyc.Max) or in an inducible manner (EREB. TCMyc), as well as in a murine fibroblast cell line (NIH3T3) overexpressing c-MYC, the team’s work showed that NBS1 was a direct transcriptional target of the c-MYC transcription factor. Furthermore, RNA interference experiments in this study confirmed the upregulation of *NBS1* expression by endogenous c-MYC in kidney epithelial cell lines (293T) and in the HeLa cancer line. Chiang et al. also reported in this study that regulation of NBS1 by c-MYC required an e-box site in intron 1 of the *NBS1* gene; and that overexpression of *NBS1* induced cell proliferation (in Rat1a line cells).

Overall, there are several indications of c-MYC involvement in the induction of DNA lesions, and this is likely correlated with its expression level, meaning that oncogenic transformation, which turns on new origins of replication and increases transcription, combined with *MYC* overexpression, could be synergistic and lead to genetic rearrangements.

## 7. Discussion: Proposed Mode of Action of c-MYC Promoting Sµ-3′RR IGH Locus Rearrangement in CLL

Genomic instability is one of the hallmarks of cancer. Reciprocal chromosome translocations are well-known in B lymphomas as a primary event in lymphomagenesis [90]. In CLL, without being frequent, chromosome translocations are not rare, occurring in 20% of cases [91], and 4 to 9% involve *IG* loci, mainly *IGH* [92]. The prognosis of CLL is then rather pejorative. It has been shown that the cytogenetic abnormalities characteristic of tumoral B cells in CLL are linked to the accumulation of DNA damage with DSBs within these cells [93]. Indeed, translocations originate from illegitimate recombination between two chromosomes as a result of DSB repair errors. Most DSBs are normally generated during physiological processes. This is the case for DSBs at the IG loci produced by RAG enzymes during V(D)J rearrangements or induced by Activation-Induced cytidine Deaminase (AID) during Ig class recombination. The DSBs can also be generated randomly as accidents of normal cellular mechanisms or following DNA damage by endogenous or exogenous agents. Unscheduled DSBs are increased by oncogenic stress due to increased rates of replication and transcription and a rise in oxidative stress leading to the production of ROS, which has been shown to be involved in the direct and indirect production of DSBs [94,95]. We observed an increase in *IGH* locus transcription in CLL cases with high *MYC* expression [7], and CLL cells have increased ROS production [96,97]. In these cases, c-MYC could participate in the genomic instability and specifically potentialize *IGH* locus recombination. Intra-*IGH* constant region recombination remains to be elucidated. Generally, in B lymphomas, translocations involving *IGH* usually result in altered expression of the partner gene, frequently an oncogene, but in CLL, the deregulation induced by these translocations is poorly understood. Thirty to forty partner loci have been listed and, in most cases, without identification of the genes involved. However, the spatial conformation of the *IGH* locus in B-cells can account for the bias of increased recombination within the *IGH* locus constant region. Recombination events depend on interactions between distantly located DNA ends that can occur because of the three-dimensional (3D) chromatin architecture. In B-cells, it has been shown the *IGH* constant region is poised in a chromatin loop holding the enhancers Eμ and 3′α, which are separated by more than 100 kb, in close proximity [98]. This structure facilitates contact between Sµ, which is proximal to Eµ, and 3′α and enables recombination between these DNA regions after DSB lesions. Whether the IGH spatial conformation is altered or not and how it favors Sµ-3′RRrec in CLL remain to be determined. Another intriguing question is the impact of such IGH recombination on the homeostasis of the tumoral CLL cell pool. By abrogating IGH expression and BCR expression at the B-cell surface, recombination of the IGH locus between the switch µ (Sµ) region and the 3′ regulatory regions (3′RR) [99,100,101,102], (Sµ-3′RRrec), is supposed to induce cell death after BCR-mediated survival signaling suppression. In our study, tumor cells from CLL Sµ-3′RRrec High cases all expressed IGM at the cell surface. The Sµ-3RRrec junctions we detected were most likely amplified from the non-functional IGH allele. Nevertheless, it can be assumed that this recombination can also target the functional allele, leading to CLL apoptosis. The study conducted was performed on biological material extracted from blood mononuclear cells, which are resting cells, and Sµ-3′RRrec detection in secondary lymphoid organs where CLL cells can be activated within proliferative centers and where a direct effect of Sµ-3′RRrec could be observed on the CLL cell pool homeostasis would be of great interest.

## 8. Conclusions

c-MYC oncogenic activity is well established concerning the activation of cell proliferation through transcription of target genes and alteration of cell cycle control which are among its classical functions. However, c-MYC appears to have non-classical functions, such as induction of DNA lesions and genetic recombination. Whether these functions contribute to tumor transformation and evolution is difficult to evaluate, but evidence of increased c-MYC expression associated with active IGH locus recombination in CLLs of poor prognosis suggests that these non-classical functions c-MYC promote more aggressive tumorigenesis. This can be achieved through genome instability. On the other hand, c-MYC-induced Sµ-3′RRrec on the IGH locus, inducing BCR loss at the cell surface, potentially influences the CLL B-cell fate. Therefore, the impact of non-classical c-MYC functions on the maintenance of genomic stability appears crucial in tumor genesis. Future studies could address how high levels of c-MYC modulate chromatin architecture and impact cellular transcriptional control in CLL. This modulation affects long-range genomic interactions, enabling the cell to coordinate the expression of genes located far apart on the genome, thereby facilitating changes in transcriptional programs. In the context of neoplastic transformation, this chromatin reorganization likely contributes to the deregulated gene expression that underpins cancer aggressiveness. Thus, the identification of MYC-specific alterations in gene expression in CLL could participate in the development of targeted therapies.

## Figures and Tables

**Table 1:** c-MYC plays a crucial role in regulating B-cell proliferation and survival, but its effects vary depending on the B-cell’s developmental stage. In pro-B cells, c-MYC regulates the cell cycle, driving pro-B cell proliferation and promoting V(D)J recombinations at the *IGH* locus. In pre-B cells, c-MYC promotes pre-B cell expansion, allowing the cells to divide before Ig light chain V(D)J recombination. As B cells mature and leave the bone marrow, *MYC* expression turns off. Once B cells recognize antigens, *MYC* expression is induced after BCR engagement to drive the rapid proliferation necessary for an effective immune response, and in the germinal center (GC), *MYC* is induced in centroblasts in the dark zone of the GC, where proliferation and somatic hypermutation occur to produce high-affinity antibodies.

Compartment	Bone Marrow		Peripheral Blood		Secondary Lymphoïd Organs			Peripheral Blood	Bone Marrow
					Extrafollicular Zone	Germinal Center			
						Dark Zone	Light Zone		
B cell Developmental Stage	Pro-B	Pre-B	Transitional B Cell	Mature B Cell	Activated B Cell	Centroblast	Centrocyte	Memory B Cell	Plasma Cell
MYC Expression	+	+	-	-	+	+	-	-	-
